# Long term disease burden post-transplantation: three decades of observations in 25 Hurler patients successfully treated with hematopoietic stem cell transplantation (HSCT)

**DOI:** 10.1186/s13023-020-01644-w

**Published:** 2021-01-31

**Authors:** N. Guffon, M. Pettazzoni, N. Pangaud, C. Garin, G. Lina-Granade, C. Plault, C. Mottolese, R. Froissart, A. Fouilhoux

**Affiliations:** 1 Reference Center for Inherited Metabolic Disorders, Femme Mère Enfant Hospital, Hospices Civils de Lyon, 59 boulevard Pinel, 69677 Bron Cedex, France; 2 Biochemistry and Molecular Biology and Reference Center for Inherited Metabolic Disorders, Hospices Civils de Lyon, 59 boulevard Pinel, 69677 Bron cedex, France; 3 Cardiology, Louis Pradel Hospital, Hospices Civils de Lyon, 59 boulevard Pinel, 69677 Bron Cedex, France; 4 Orthopaedy, Femme Mère Enfant Hospital, Hospices Civils de Lyon, 59 boulevard Pinel, 69677 Bron Cedex, France; 5 Oto-Rhino-Laryngology and Child Audiology, Femme Mère Enfant Hospital and Edouard Herriot Hospital, Hospices Civils de Lyon, 59 boulevard Pinel, 69677 Bron Cedex, France; 6 Neurosurgery, Femme Mère Enfant Hospital and Pierre Wertheimer Hospital, Hospices Civils de Lyon, 59 boulevard Pinel, 69677 Bron Cedex, France

**Keywords:** Mucopolysaccharidosis type I-Hurler syndrome, Hematopoietic cell transplantation, Long-term outcomes, Residual disease burden, Depression, Psychosocial function, Adaptation, Cognitive

## Abstract

**Background:**

Mucopolysaccharidosis type I-Hurler syndrome (MPSI-H) is a lysosomal storage disease characterized by severe physical symptoms and cognitive decline. Early treatment with hematopoietic cell transplant (HSCT) is critical to the survival of these patients. While survival rates and short-term outcomes are known to be improved by HSCT, the long-term cognitive, adaptive and psychosocial functional outcomes of children with (MPSI-H) post-HSCT are not well documented. This manuscript focuses on retrospective long-term follow-up (7–33 years) of 25 MPSI-H patients, transplanted between 1986 and 2011.

**Results:**

The median age at transplantation was 21 months (range 12–57 months). Except for one death, all successfully transplanted MPSI-H patients surviving at least 1 year after HSCT are alive to-date, with a median age of 21 years (range 8–36 years) at the last follow-up evaluation. A majority of HSCT grafts were bone marrow transplants (BMT), resulting in durable full chimerism in 18 (72%). Pre-HSCT, the onset of first symptoms occurred very early, at a median age of 3 months (range birth-16 months). The most prevalent symptoms before MPSI-H diagnosis involved progressive dysostosis multiplex; almost all patients suffered from hip dysplasia and thoracolumbar spine Kyphosis. Despite HSCT, considerable residual disease burden and ensuing corrective surgical interventions were observed in all, and at every decade of follow-up post HSCT. Late-onset psychiatric manifestations were significant (n = 17 patients; 68%), including depression in 13 patients at a median onset age of 18 years (range 13–31 years), hyperactivity and attention deficit disorder (n = 4), and multiple acute psychotic episodes (APE), independent of depression observed (n = 3) at a median onset age of 18 years (range 17–31 years). The adult Welscher Intelligence Scale results (n = 16) were heterogenous across the four scale dimensions; overall lower scores were observed on both working memory index (median WMI = 69.5) and processing speed index (median PSI = 65), whereas verbal comprehension index (median VCI = 79) and perceptual reasoning index (median PRI = 74) were higher.

**Conclusion:**

With advanced treatment options, MPSI-H are living into 3rd and 4th decades of life, however not disease free and with poor adaptation. Residual disease (loss of mobility, limited gross and fine motor skills; low cognitive ability; suboptimal cardiopulmonary function, vision and hearing) negatively impacts the quality of life and psychosocial functioning of affected individuals.

## Background

Mucopolysaccharidosis I (MPS I) is a rare autosomal recessive lysosomal storage disorder associated with the accumulation of glycosaminoglycans (GAGs: heparan and dermatan sulfate substrates) in tissues and organs due to a deficiency of α-l-iduronidase (IDUA). The accumulation of these substrates leads to progressive multisystem disease with a wide range of clinical manifestations and a continuum of severity [1–3]. With an estimated incidence of 1:100,000 live births, MPS I is categorized as either of 3 forms according to age at onset, clinical severity, and rate of developmental deterioration. Hurler syndrome (MPSI-H) is the most severe and most prevalent (50–80%), whereas Hurler–Scheie (MPSI-HS) and Scheie syndromes (MPSI-S) are more attenuated forms of MPS I [4].

Over 200 pathogenic IDUA variants have been identified underlying the MPS I disorder, and there appears to be a close genotype–phenotype correlation with severe disease. Patients who are either homozygous or compound heterozygous for two variants predictive of severely disrupting gene transcription or translation (eg, nonsense variants, frameshifts, consensus splice disruption or initiator codon variants) consistently express severe disease. The more common nonsense mutations are W402X or Q70X [5]. There is however no biochemical assay to assess or classify patients as either severe or attenuated.

The natural course of disease in the MPSI-H begins at birth or shortly after during the first few months of life. There are notable changes in the physical appearance, frequent rhinitis and ear, nose and throat (ENT) infections, respiratory obstruction and sleep apnea, heart valve disorders, frequent hernias (inguinal and umbilical), progressive visual impairment (corneal clouding, cataracts, glaucoma, retinal degeneration), hearing impairment or deafness, delayed neurocognitive developmental skills, and dysostosis multiplex (hip dysplasia, thoracolumbar kyphosis, genu valgum, enlarged skull, spatulate ribs, thickened diaphysis, and bullet shaped metacarpals), compromised growth, limited joint mobility and nerve entrapment syndromes (e.g. cervical or spinal cord compression and carpal tunnel syndrome) [6–9]. Clinical manifestations gradually worsen with age. If left untreated, the life expectancy of an MPSI-H patient is less than 10 years with cause of death most commonly due to severe respiratory insufficiency and cardiopulmonary disease, upper respiratory infections or cardiac manifestations [4].

The standard of care for patients with MPSI-H is treatment with hematopoietic stem cell transplantation (HSCT), and in combination with palliative and symptoms-specific treatments tailored to meet the functioning needs as well as overall quality of life of patients. Bone marrow transplant (BMT), has been used in the treatment of MPSI-H since 1980s, whereas unrelated cord blood transplant (CBT) has become more widely acceptable [10–13].

Enzyme replacement therapy (ERT) with Laronidase (available since 2003) has shown to alter somatic disease symptoms and disease progression, but as it does not cross the blood brain barrier, this treatment option alone does not address the progressive central nervous system (CNS) decline [14, 15]. ERT is often used as adjunctive therapy, in the peri transplantation period, to clear much of the storage material and to improve the health status of patients prior to HSCT, however it is not offered universally [14, 16–20].

The immediate benefits of successful HSCT, alone or in combination with ERT has demonstrated significant improvement in somatic symptoms, urinary GAG levels, enzyme levels and with less severe cognitive decline following HSCT. Longer-term benefits depend on the age and disease burden at the time of transplant and include extended life expectancy; preservation of neurocognitive and intellectual development especially if transplantation is initiated prior to the onset of neurological symptoms; improved endurance and physical activity; growth and overall quality of life [10, 12, 13, 19–26]. HSCT does not, however, adequately treat all disease manifestations, even in optimal situations of complete engraftment from enzyme-normal donors. HSCT decelerates or stabilize some somatic manifestations but does not reverse disease progression; MPSI-H patients still experience significant persisting disease burden and many continue to require multiple surgical interventions (e.g., adenotonsillectomy, hernia repair, ventriculoperitoneal shunt, orthopedic surgeries, carpal tunnel release, cerebral fluid shunts, venting tubes for recurrent otitis media, corneal grafts, cardiac valve replacement), despite successful transplantation [1, 10–12, 18, 19, 22, 23, 25–32].

Important contributions to the understanding of the efficacy and safety of HSCT and ERT and the residual disease burden post transplantation have been documented in the literature. However, most studies conducted have been limited either in sample size or duration of follow-up or have focused on one or few of the somatic disease manifestations. The very long-term cognitive, adaptive and quality of life outcomes of MPSI-H patients post-transplant are not well understood, and there is a paucity of literature surrounding psychiatric events (depression, psychotic episodes, dementia, hyperactivity) and long-term psychosocial outcomes, especially social adaptation and social functioning given the long-term survival rates.

The aim of this publication is to describe the long-term residual disease burden and the current psychosocial adaptation and functioning of a cohort of 25 MPSI-H patients successfully transplanted with HSCT and followed for over a maximum of a 32 years period at the Reference Center for Inherited Metabolic Disorders (RCIMD), Lyon, France. Through this publication, we aim to provide health care professionals with better insight into the MPSI-H patients’ psychosocial adaptation, especially with respect to residual disease burden post HSCT.

## Results

This manuscript focuses on long term follow-up of patients, therefore only successfully transplanted MPSI-H patients (those surviving 1-year post transplantation) were included in the analysis. Results presented reflect the retrospective analysis of long-term follow-up evaluations (7–33 years) of 25 MPSI-H patients, transplanted between 1986 and 2011.

### Patient Characteristics

A total of 25 MPSI-H patients (10 male and 15 female) underwent 28 HSCT at a median age of 21 months (range 12–58 months) (Table 1). Except for one death, all MPSI-H patients are alive to-date with median age of 21 years (range 8–36 years) at the last follow-up evaluation. The median follow-up time was 20 years (range 7–33 years) post HSCT. One patient died at 17 years of age (15 years post HSCT) due to cardiac arrest following cardiopulmonary complications and respiratory failure. A majority (23 of 25) of patients are Caucasian; two patients are of North African descent. Three (unrelated) patients are the offspring of consanguineous marriages between first cousins. The median age at MPSI-H diagnosis was 10 months (range 2–34 months). In all patients, diagnosis was confirmed by increased urinary GAGs as well as deficiency of IDUA activity in leukocytes [6]. All codon and the immediately flanking intronic regions of the IDUA gene were sequenced. In all patients, mutation analysis revealed a genotype associated with severe phenotype and most (15 patients) had mutations p.W402X or p.Q70X on one or both alleles (Table 1).

**Table 1:** Patient Characteristics

	n	(%)	Median	(Range)
Patient characteristics				
Sex (male/female)	10/15	(40/60)		
Ethnicity (caucasian)	23	92		
Donor characteristics				
Source (CB/BM)	3/22	(12/88)		
Relation (related)	8	(32)		
Carrier status (carrier)	6	(24)		
Transplantation characteristics				
Number of transplantations (1/2/3)^a^	23/1/1	(92/4/4)		
Year of transplantation			1999	(1986–2011)
Post-HSCT ERT/peri-transplantation ERT	5/3	(20/12)		
Donor chimerism (full)	18	(72)		
HLA (identical)	14	(56)		
Post-transplantation				
GVHD acute	9	(36)		
GVHD chronic	0	(0)		
Alopacie	3	(12)		
Infection (viral/bacterial)	4/4	(16/16)		
Death*	1	(4)		
Genotype				
W402X/W402X	9	(36)		
W402X/Q70X	6	(24)		
W402X/Y581X	1	(4)		
W402X/134del12	1	(4)		
W402X/c.50_61del12	1	(4)		
W402X/1124 del C	1	(4)		
Q70X/c.792 + 1G > A in intron 5	1	(4)		
Q70X/c.1190-10C > A	1	(4)		
Q70X/A327P	1	(4)		
P533R/c.1273_1274insC	1	(4)		
1041_1042 del CA/1041_1042 del CA	1	(4)		
ND in USA	1	(4)		
Follow-up post HSCT (years)				
7–10	3	(12)		
11–15	3	(12)		
16–20	10	(40)		
21–25	5	(20)		
25+	4	(16)		
Age at last follow-up (years)			19	[8, 36]
Age onset of symptom (months) [range]			3	[0–16]
Age diagnosis (months) [range]			10	[2, 34]
Age HSCT (months) [range]			21	[12, 57]

*Transplantation Characteristics* ^a^one patient was transplanted a second time following graft failure from donor father; a second patient recieved a 3rd BMT after two graft failures from the same unknown donor; *One patient died at the age of 17 years (15 years post HSCT) due to cardiac arrest following cardiopulmonary complications and respiratory failure

*CB* cord blood, *BMT* bone marrow transplantation

### Peri-engraftment

A majority of HSCT grafts were bone marrow transplants (BMT): 25 BMTs were performed in 22 patients (Table 2). Within the first year post-HSCT, full donor chimerism (> 95%) was achieved in a majority (n = 19, 76%); durable full engraftment was observed in 18 patients (75%). Seven patients had mixed chimerism (< 90%) (2 patients with chimerism between 80 and 90% and 5 patients with chimerism decreased below 50% from 2 years post HSCT and were subsequently treated with long term ERT between 1.5 and 24 years after HSCT.

**Table 2:** Engraftment, genotype severity, IDUA activity, chimerism, ERT, GVHD

ID	Genotype	Quantitative Urinary GAG mg/g creatinine		IDUA, % of day control^^^^		ERT	Age at HSCT (months)	Donor type			Cell dose		Chimerism post-transplantation			
		Initial	Current^^^^	Leukocytes [ukat/kg] [NR: 3.4–11.8]	Serum [nkat/l] [NR: 1.50–4.9]					HLA	TNC/kg	GVHD	1 month (%)	2 years (%)	5 years (%)	Current^^^^ (%)
1	W402X/Q70X	232 DS++ HS +	6.2 DS- HS-	[10.3] 100%	[1.70] 85%	No	14	BMT	HO sibling	10/10	1.3	C, GI	> 95	> 95	> 95	> 95
2	Q70X/c.1190-10C > A	184 DS++ HS+	7.1 DS++ HS± (pré-ERT 14 DS++ HS +)	[12.3] 112% (pre-ERT [5.7] 79%)	[0.50] 25% (pre-ERT [0.12] 10%)	24 y post HSCT	58	BMT	HO sibling	10/10	3.2		> 95	40	50	34
3	W402X/134del12	177 DS++ HS+	5.0 DS+ HS+	[10.2] 94%	[1.60] 80%	No	42	BMT^u^		9/10 MM A	4.7	C, D, GI	> 95	> 95	> 95	> 95
4	W402X/W402X	247 DS+++ HS+	4.8 DS+ HS+	[4.1]43%	[0.50] 22%	No	14	BMT	HET father	10/10	9.2		> 95	> 95	> 95	> 95
5	1041_1042 del CA/1041_1042 del CA	179 DS+++ HS+	4.8 DS+ HS±	[7.1] 79%	[1.74] 33%	No	42	CB	HET sibling	10/10	0.7	H, GI	54	88	88	85
6	1124 del C /W402X	181 DS+++ HS+	7.9 DS± HS± (pre-ERT 29 DS+ HS-)	[5.3] 84% (pre-ERT 2.2 30%)	[0.26] 0.1% (pre-ERT [0.6] 0.1%)	7 y post HSCT	23	BMT^u^		7/10 MM B, 2Cw	9.1		95	20	10	59^**^
7	W402X/Q70X	231 DS++++ HS++	8.2 DS+ HS+	[6.9] 66%	[0.44] 15%	No	14	BMT	HET sibling	10/10	4.0		> 95	> 95	> 95	> 95
8	c.50_61del12/W402X	116 DS+++ HS+	4.6 DS+ HS+	[12.1] 102%	[1.04] 37%	No	21	BMT^u^		10/10	8.0		> 95	> 95	> 95	> 95
9	W402X/W402X	195 DS+++ HS+	5.1 DS± HS±	[8.4] 80%	[2.46] 84%	No	25	BMT^u^		7/10 MM A, 2Cw	5.3		80	50	60	82
10	W402X/W402X	198 DS++++ HS+	5.2 DS± HS±	[10.9] 116%	[1.8] 107%	No	26	BMT^u$^		8/10 MM A, Cw	5.0		> 95	> 95	> 95	> 95
11	Q70X/W402X	94 DS+++ HS++	4.6 DS± HS+	[7.7] 90%	[1.10] 49%	No	16	UBMT		10/10	6.0		> 95	> 95	> 95	> 95
12	W402X/Q70X	121 DS+++ HS++	5.1 DS+ HS± (pre-ERT 9.6 DS++ HS±)	[7.6] 63% (pre-ERT [3.4] 29%)	[1.06] 32% (pre-ERT [0.43] 22%)	14.5 y post HSCT	20	BMT^u^		7/10 MM A, 2Cw	8.4		100	40	68	70
13	Q70X/A327P	108 DS++++ HS+++	3.9 DS± HS± (pre-ERT 24.7 DS++ HS±)	[11.5] 100% (pre-ERT [3.8] 44%)	[1.54] 44% (pre-ERT [0.6] 6%)	6 y post HSCT	35	BMT	HET sibling	10/10	4.5		NC	50	30	52
14	W402X/W402X	169 DS+++ HS++	4.3 DS± HS-	[12] 115%	[0.71] 22%	No	25	BMT^u^		7/10 MM A B DRB4	10.6		> 95	> 95	> 95	> 95
15	P533R/c.1273_1274insC	116 DS+++ HS++	3.9 DS+ HS+	[8.8] 70%	[1.41] 52%	No	23	BMT^u^		7/10 MM B C DQB1	7.3		> 95	> 95	> 95	> 95
16	W402X/W402X	182 DS+++ HS++	4.9 DS± HS±	[6.5] 63%	[0.57] 36%	No	12	BMT	HET sibling	10/10	6.2	C, D, GI	> 95	> 95	> 95	> 95
17	W402X/Y581X	167 DS+++ HS++	3.2 DS+ HS±	[14.8] 117%	[1.62] 60%	No	21	BMT^u^		7/10 MM A B Cw	9.7	D, GI	> 95	> 95	> 95	> 95
18	W402X/W402X	150 DS+++ HS++	5.7 DS+ HS±	[3.5] 37% (pre-ERT [0.8] 20%)	[0.30] 13% (pre-ERT [0.04] 0.01%	1.6 y post HSCT	18	BMT^u^		10/10	9.1		75	35	44	47
19	Q70X/W402X	high level*	4.5 DS+ HS+	[10.1] 93%	[1.40] 70%	No	28	BMT^u$^		9/10 MM B	13.4	C, D, O, H, GI	> 95	> 95	> 95	> 95
20	W402X/W402X	110 DS++++ HS++	5.6 DS+ HS+	[12.2] 120%	[1.60] 130%	No	25	BMT^u^		10/10	9.6	C, D, GI	> 95	> 95	> 95	> 95
21	Q70X/W402X	161.5 DS++++ HS++	7.0 DS+ HS+	[7.9] 94%	[0.74] 43%	No	20	BMT^u^		10/10	5.4		> 95	> 95	> 95	> 95
22	W402X/W402X	80	7.1 DS+ HS+	[9.2] 84%	[1.10] 55%	peri-HSCT (10 months)	31	BMT^u^		10/10	8.2	C, D, GI	> 95	> 95	> 95	> 95
23	Q70X/c.792 + 1G > A in intron 5	241	20.8 DS+ HS±	[4.3] 40%	[0.50] 19%	peri-HSCT (4 months)	13	BMT	HET sibling	10/10	4.3		> 95	> 95	> 95	> 95
24	ND in USA	high level* (USA)	10.9 DS+ HS±	[10.0] 105%	[1.20] 52%	No	12	CB^u^		4/6 MM A, B	1.2	C, GI	> 95	> 95	> 95	> 95
25	W402X/W402X	156	ND	[13.9] 124%	[0.80] 31%	peri-HSCT (6 months)	13	CB^u^		5/6 MM A	1.5		> 95	> 95	> 95	> 95

^^^^At most recent evaluation; ^$^reporting only the successful (last) transplantation; *NR* normal range, *BMT* bone marrow transplant; ^u^unknown source; *CB* cord blood, *HO* homozygous, *HET* heterozygous;

GAGu: [Normal range 8–29 mg/g creatinine (1–3 years of age); 6–23 mg/g creatinine (3–7 years of age); 3–16 mg/g creatinine (7–15 years of age); 1–15 mg/g creatinine (15–20 years of age); 1–8 (20 + years of age)]

*DS* dermatan sulphate, *HS* heparan sulfate; -, ±, +, ++,+++

*GVHD* graft versus host disease, *H* hepatic, *C* cutaneous, *D* digestive, *O* ophthalmic, *GI* gastrointestinal

^**^Prior to death; NC = non quantifyable; ND = not done; *different assay used

A total of nine (36%) patients developed acute graft versus host disease (GVHD) (grade I/II), including cutaneous, hepatic, ocular and digestive involvement, for which symptoms were resolved with corticosteroids and inolimomab (Table 2). There were no incidents of chronic GVHD. One patient suffered from severe haemolytic anemia that resolved with corticosteroid treatment after tapering cyclosporine. Three patients suffered from long-term irreversible alopecia secondary to Busulphan conditioning complications.

### Chronology of symptoms onset

**Fig. 1:**
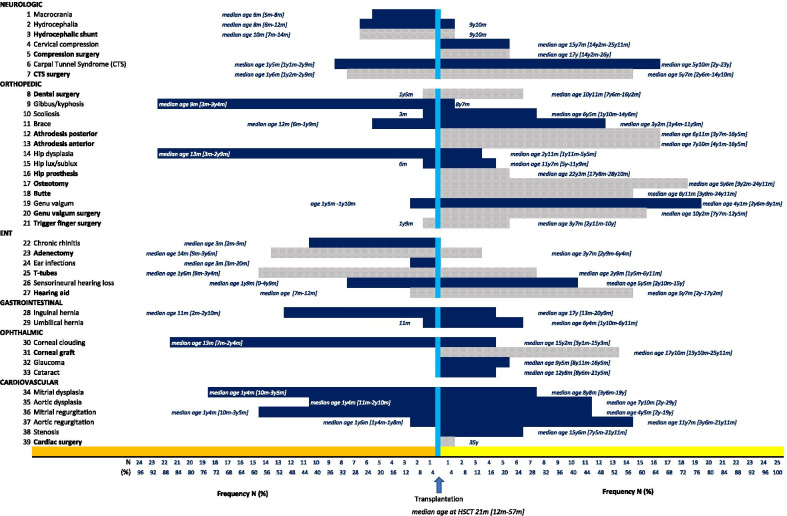
Age at various disease manifestation onsets and surgical interventions with respect to time of transplantation (HSCT) in 25 patients with Mucopolysaccharidosis I Hurler (MPSI-H). The median age at HSCT was 21 months of age. The horizonal frequency distributions of disease manifestation onset (darked shaded bars) and surgical interventions (lighter shaded bars) are adjusted with respect to age at transplantation, such that events to the left of HSCT reflect pre-HSCT and those to the right post-HSCT

Patients were evaluated on a regular basis, at 1, 3, and 6 months, 1-year post HCT, and annually thereafter until June 2019. The rate of symptoms onset (median age at symptoms onset) with respect to transplantation (before and after HSCT), as well as the overall disease burden in this cohort of patients is presented in Fig. 1.

The onset of first symptoms occurred very early on. The median age at first symptom onset was 3 months (range birth-16 months); four patients had symptoms at birth (including metatarsus varus, arthrogryposis or neonatal hearing loss), and a further 16 (64%) patients developed symptoms before the age of 3 months.

### Skeletal/bone manifestation

The most prevalent disease manifestations appearing prior to MPSI-H diagnosis and treatment involved deformities of the skeletal system. Dysostosis multiplex progressed in all patients despite HSCT, requiring multiple and frequent surgical interventions in the majority. Thoracolumbar spine Kyphosis was observed in 24 (96%) patients presenting early at a median age of 8 months (range 3–42 months), half of them had been diagnosed with gibbus prior to MPSI-H diagnosis. Scoliosis developed in eight of these patients, manifesting at a median age of 5.3 years (range 3 months–14.5 years). A skeletal/vertebral brace was worn by 19 (76%) patients, beginning at a median age of 27 months (range 6 months–11.8 years) and for a mean duration of 8.5 ± 4.5 years (range 2–16 years). A kyphotic angle however, progressed in 17 (68%) patients, requiring arthrodesis (posterior and anterior) at a median age of 6.9 years (range 3.5–16.4 years). Three of these patients required extensive surgical revisions due to recurrence of kyphosis post-arthrodesis.

All patients suffered from hip dysplasia, with diagnosis as early as 3 months (median age of 13 months). Two patients were treated for hip luxation at 6 months of age, and before the diagnosis of MPSI-H disease. Hip dysplasia progressed with time for all patients, and eventually 23 patients underwent hip surgery. Eighteen (76%) patients underwent femoral osteotomy procedures (bilateral in 16/18) at a median age of 5.5 years (range 3.1–25 years); four involved the pelvis, and 14 had hip abutments (at median age of 7 years, range 3.9–25 years). Five patients had hip prosthesis surgery (2 bilateral, 3 unilateral) at median age 22.3 years (range 17.6–28.8 years), three of whom had had a previous femoral osteotomy and hip abutment.

Genu valgum, most often bilateral was observed in 23 (92%) patients. Worsening of genu valgum lead to epiphysiodesis surgery in 15 patients (60%) at median age of 10.2 years (range 7.6–12.4 years).

Seven (28%) patients had periodontal cysts or impacted teeth that required oral surgery; two of these patients had severely restricted mouth opening requiring coronoidectomy (median age of 10.3 years, (range 17 months–16.1 years).

### Neurologic manifestation

Hydrocephalus was observed in 6 patients pre-HSCT, requiring ventriculoperitoneal shunting at a median age of 10 months (range 7–14 months); one patient developed hydrocephaly at 10 years of age, post-HSCT and two patients developed intracranial hypertension (ICHT) due to shunt dysfunction (ages 8 years and 19 years).

Narrowing of the spinal canal was observed in all patients starting at a median age 2.8 years (range 6 months–27 years). Surgical interventions were required in six patients; in five (20%) for medullary compression at a median age of 17 years (range 14.2–26 years) and for atlantoaxial instability (C1–C2) in one patient at 14 years of age.

Carpal tunnel syndrome developed in 24 (96%) patients, most often affecting bilateral wrists (23 bilateral); eight patients developed CTS pre-HSCT at a median age of 17 months (range 13–33 months); 16 were diagnosed with CTS post-HSCT at median age of 5.8 years (range 2–23 years). Surgery to release nerve entrapment was conducted in 22 (88%) patients at a median age of 3.8 years (range 14 months–14.8 years). Other hand surgery for trigger finger or tendon relapse was performed in 6 patients (24%).

### Ophthalmic manifestation

Corneal clouding was observed in all patients. In 84% of patients the symptom was diagnosed before HSCT at a median age of 13 months (range 7–34 months). Despite HSCT. corneal clouding worsened in all patients and approximately half of the patients underwent corneal graft surgery at a median age of 17.8 years (range 13.7–25.9 years). Five patients had glaucoma, all successfully treated with anti-glaucoma therapy. Four patients had cataracts diagnosed at a median age of 12.7 years (range 8.5–21.4 years), three of whom were surgically corrected. None of the patients developed retinopathy. However, optic atrophy and irreversible blindness secondary to ICHT occurred in one patient at 19 years of age.

### ENT and auditory manifestations

Most patients (n = 22, 88%) had at least one surgical ENT intervention. A total of 18 (72%) patients underwent an average of 1.2 (range 1–3) adenectomies at a median age of 17 months (range 9 months–6.3 years). Among those with multiple adenectomies, the last procedure was performed at a median age of 7 years (range 4–10 years).

Frequent chronic otitis media led to multiple and repeated grommet insertions; twenty-one (84%) patients were fitted with 54 tympanostomy tubes (T-Tubes), often bilaterally; for the first of whom was placed at median age of 20 months (range 9 months–6.9 years) and the last of whom was fitted at median age 9 years (range 2–27 years). Tympanoplasty was performed in 3 patients with perforated tympanic membranes.

Sensorineural hearing impairment was observed in 18 patients (72%) and was most often bilateral (n = 13), at a median age of 3 years (range birth-15 years). A third of the patients had some hearing impairment before HSCT. Long-term hearing appeared to stabilize in most after HSCT, ranging in intensity from mild (26–40 dB) in 10 patients, to moderate (40–55 dB) in 5 patients, and moderate-severe (56–90 dB) in 3 patients. Although hearing aids were prescribed, they were not always well-accepted; only half were wearing them as adults.

### Cardiopulmonary manifestation

The progression of cardiovascular disease was observed in all patients. None of the patients developed electrocardiogram abnormalities. Valve thickening was observed in 20 patients before HSCT involving the aortic valve (n = 2), mitral valve (n = 10), or both mitral and aortic valves in 8 patients, at a median age of 16 months (range 10 months–3.4 years). A total of 16 patients developed valve regurgitation pre-HSCT (14 mitral and 2 aortic regurgitation), and for whom worsened in 7/14 and 2/2 respectively. A further 11 and 14 patients developed mitral regurgitation or aortic regurgitation post-HSCT at a median age of 4.4 years (range 2–19 years) and at a median age of 11.6 years (range 3.5–21.9 years), respectively. Regurgitation continued to worsen in three patients. Seven patients with mitral dysplasia pre-HSCT developed an aortic dysplasia, and the 2 patients with aortic dysplasia pre-HSCT developed mitral dysplasia.

Six patients developed a valve stenosis by the median age of 15.5 years (range 7.4–21.9 years), necessitating a valve replacement in one (35 years of age), and surgical intervention for abdominal aortic coarctation in a second patient at the age of 14 years.

**Fig. 2:**
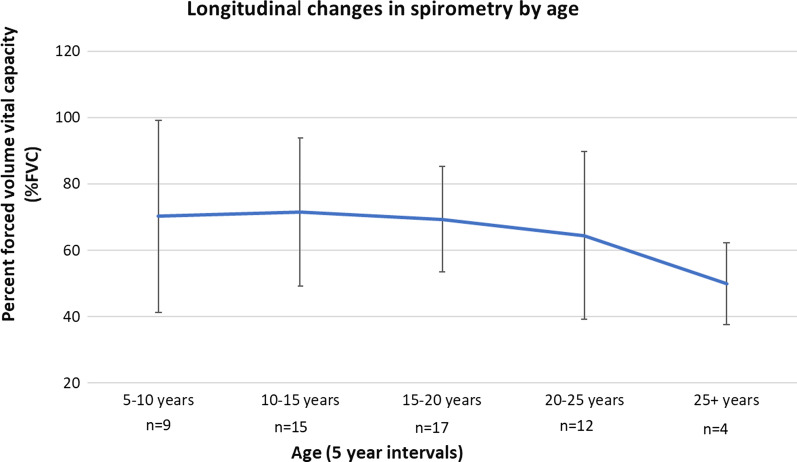
Longitudinal change in spirometry by age. Pulmonary function as assessed by spirometry [percent forced volume vital capacity (% FVC)] was impaired but largely stable for most; only 3 (12%) patients achieved normal FVC > 80%. Although there was no indication of obstructive syndrome, 15 (60%) had signs of restrictive syndrome on last evaluation with median FVC of 63% (range 38–79%). Six patients (24%) were unable to perform pulmonary function test on their last follow-up evaluation because of severe cognitive impairment. Note be taken that the youngest age for spirometry evaluation was 6 years

Pulmonary function (Forced vital capacity, FVC) was assessed at each follow-up (Fig. 2). Only 3 (12%) patients achieved normal FVC > 80% and 15 (60%) had signs of restrictive syndrome on last evaluation with median FVC of 63% (range 38–79%). Spirometry outcomes indicate impaired but largely stable pulmonary disease until the age of 20 years, and a decline thereafter. In 5 patients receiving long-term ERT because of partial engraftment, there was no difference seen in FVC% as compared to the rest of the cohort.

### Anthropometric (height)

**Fig. 3:**
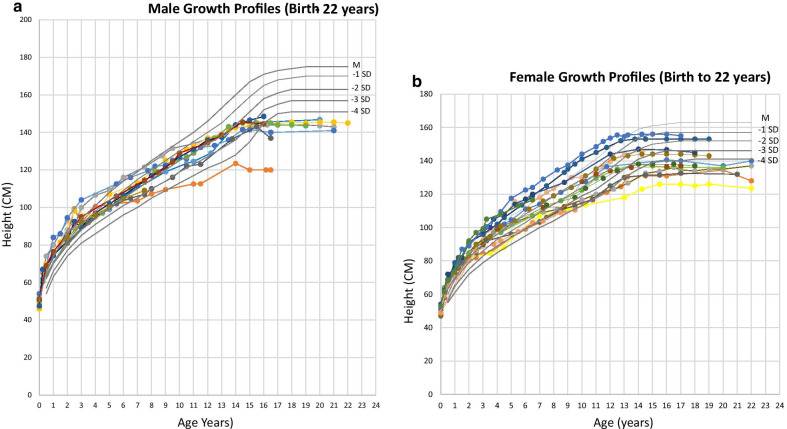
Growth profiles of male (n = 10) and female (n = 15) patients with MPSI-H as compared with sex-specific French growth reference charts. *SD* standard deviation; **b**. Growth plots of 15 female patients shows normal growth velocity for approximately a third of the patients until the age of 14; two thirds of the patients had growth velocities below -1SD, one third remained below -2SD. Most female patients crossed the -1SD curve around the age of 11 years and -2SD curve by 13 years of age (around the age of puberty). There was a suboptimal pubertal growth spurt (between 6 and 15 cm as compared to the expected 25–28 cm), followed by growth cessation, as compared with sex-specific French growth reference charts. Thirteen (87%) female patients reached menarche at a median age of 13 years (range 11–15 years), albeit, all female patients demonstrated either premature ovarian failure or ovarian insufficiency. **a**. Although linear growth for males was maintained for several years, growth velocity fell to lower than -2SD around the age of puberty in all male patients. Puberty was normal for most, except for one patient who received therapy for premature puberty. The final adult height for male patients ranged from 120 to 145 cm (median height 142.5 cm), and target adult height was never reached

Growth was compromised at all ages, the final adult height for 22 patients ranged from 124 to 155 cm for females and 120–145 cm for males (median height of 142.5 cm for both males and females alike) (Fig. 3a, b).

### Neurodevelopmental function

Neurodevelopmental function was measured at baseline (prior to grafting) and on multiple follow-up evaluations post-HSCT (Table 3). All children made cognitive progress initially, before plateauing and then progressively declining. Even though neurocognitive deterioration stabilized from 1 year post-HSCT and new skill gains and learning were observed in children (three obtained high school vocational diplomas), most of the patients demonstrated long-term mild to moderate cognitive impairment in all domains of assessment. Baseline cognitive function indicated mild impairment (scores < 80) in seven patients (28%).

**Table 3:** Long-term evaluation of neurocognitive development among 25 MPSI-H patients

Case ID	Genotype	Sex	Current age	Age at HSCT (months)	Mean QD scores (Years of age)					WISC III or IV (8–10 years of age)					WISC III or IV (10–13 years of age)					WISC IV (13–17 years of age)					WAIS IV (17 + years of age)				
					Baseline	1–2 years	2–4 years	4–6 years	6–8 years	VCI	PRI	WMI	PSI	FSIQ	VCI	PRI	WMI	PSI	FSIQ	VCI	PRI	WMI	PSI	FSIQ	VCI	PRI	WMI	PSI	FSIQ
1	W402X/Q70X	f	34	14	75	77		97							VIQ 103				95						100	66	71	66	71
2	Q70X/c.1190-10C > A	m	37	58	95			95	90					90	VIQ 87	PIQ 92			88						86	78	58	64	67
3	W402X/134del12	f	32	42	77		77	90	100	VIQ85				85											73	88	77	89	77
4	W402X/W402X	f	27	14	100	80	83	83	84	VIQ82	PIQ71			73	VIQ 72	PIQ75			70	80	60	60	55	53	77	58	55	58	53
5	1041_1042 del CA/1041_1042 del CA	f	24	42	70		70												30					< 30					< 30
6	1124 del C /W402X	m	–	23	94	94	72	90	84					82					77					53	DCD	DCD	DCD	DCD	DCD
7	W402X/Q70X	m	23	14	100	100	99												84						69	64	61	58	55
8	c.50_61del12/W402X	m	24	21	90	90	73	80	77					68					67	63	102			64	65	69	55	54	61
9	W402X/W402X	f	24	25	70	70	91	80	73					58					46					35					< 30^b^
10	W402X/W402X	f	23	26	100	100	85	93	85	R	R	R	R	R	88	69	64	50	61	B	B	B	B	B	B	B	B	B	B
11	Q70X/W402X	f	22	16	93	93	100	93	97	98	81	79	86	80					80					72	86	74	74	61	69
12	W402X/Q70X	m	22	19	83	81	88	82	79					79					73						73	74	77	50	65
13	Q70X/A327P	f	22	34	82	82	89	82	62					59					52					30					< 30
14	W402X/W402X	f	21	25	80	80	69	77	89					88	92	88	115	112	100	86	84	115	93	90	89	87	88	87	80
15	P533R/c.1273_1274insC	f	20	23	88	87	84	96	87					87					88					76	79	84	94	81	79
16	W402X/W402X	f	19	12	80	80		100	100						101	82	109	86	92					82	84	78	83	72	75
17	W402X/Y581X	m	20	31	94		93	86	85					84					76	103	70	80	92	79	84	62	83	75	70
18	W402X/W402X	m	19	18	86	91	79	77	75					69					58					50	61	68	66	50	52
19	Q70X/W402X	f	20	28	80	81	83	80	77	69	67	56	69	53					71	72	69	76	81	66	79	66	68	69	63
20	W402X/W402X	f	19	25	86	86	95	78	76	79	96	62	83	75	94	79	58	81	73					70	75	82	66	75	69
21	Q70X/W402X	m	17	20	79	79	79	82	80	108	88	70	76	83	86	79	62	66	68						79	86	68	58	69
22	W402X/W402X	m	17	31	70		70	60	46					40					40	45	45	50	50	40					NA
23	Q70X/c.792 + 1G > A in intron 5	f	10	13	77	83	96	94	95					93	96	81	91	93	86					NA					NA
24	ND in USA	m	9	12	100	100				96	94	70	90	86					NA					NA					NA
25	W402X/W402X	f	8	13	88	66	50^a^	< 30	< 30					< 30					NA					NA					NA
			N		25	20	21	22	21	7	6	5	5	20	9	8	6	6	22	6	6	5	5	15	16	16	16	16	19
			Mean		85.5	85.0	83.8	85.5	82.1	90.0	85.2	67.4	80.8	75.4	92.8	79.7	83.2	81.3	71.6	74.8	71.7	76.2	74.2	61.4	78.7	74.0	71.5	66.7	67.2
			std		9.8	9.4	10.0	9.4	12.8	15.7	11.7	8.8	8.3	14.3	5.5	6.2	25.3	21.5	18.1	19.9	19.6	24.8	20.4	18.4	9.7	9.6	11.6	12.3	8.7
			Median		86.0	82.5	83.5	83.0	84.0	96.0	88.0	70.0	83.0	80.0	93.0	80.0	77.5	83.5	73.0	76.0	69.5	76.0	81.0	65.0	79.0	74.0	69.5	65.0	69.0
			Min		70.0	66.0	69.0	60.0	46.0	69.0	67.0	56.0	69.0	40.0	86.0	69.0	58.0	50.0	30.0	45.0	45.0	50.0	50.0	30.0	61.0	58.0	55.0	50.0	52.0
			Max		100.0	100.0	100.0	100.0	100.0	108.0	96.0	79.0	90.0	93.0	101.0	88.0	115.0	112.0	100.0	103.0	102.0	115.0	93.0	90.0	100.0	88.0	94.0	89.0	80.0

*PIQ* performance index, *VIQ* verbal intellectual quotient, *VCI* verbal comprehension index, *PRI* perceptual reasoning index, *WMI* working memory index, *PSI* processing speed index, *FSIQ* full scale intelectual quotient

^a^Autism diagnosed at 2 years of age

^b^Dimentia diagnosed at 19 years of age; *R* refused, *B* blind, *DCD* deceased, *NA* not applicable

Five patients (case 5, 9, 13, 22 and 25) suffered from severe cognitive impairment (IQ less than 40) which was detected as early as 4–13 years of age, and consequently were not able to complete all domains of neurodevelopmental tests. Three of these patients (case 5, 13 and 22) were more likely to have HSCT at an older age (after the age of 30 months) as compared to the rest of the cohort (3/5 (60%) versus 2/20 (10%) respectively), three had an IQ of 70 (case 5, 9, 22) before HSCT, three had variable chimerism (case 5, 9, 13) as compared to the rest of the cohort [n = 4/20 (20%)], and three (case 9, 22, 25) had developed psychiatric troubles (hyperactivity, autism, and dementia).

The Wechsler intelligence scale assessed in adults (WAIS IV, 17+ years of age) results were heterogenous across the four scale dimensions. On last evaluation, the patient cohort (n = 16) demonstrated overall lower scores on both working memory index (median WMI = 69.5; n = 16) and processing speed index (median PSI = 65), whereas verbal comprehension index (median VCI = 79) and perceptual reasoning index (median PRI = 74) were higher.

### Scholastic achievement

All children were initially placed in the mainstream educational system and supervised in special education classes. Throughout schooling years, a speech therapist evaluated each child for age-appropriate scholastic and social functioning at each follow-up visit. The highest scholastic level attained for each patient is listed in Table 4. On last evaluation, six (24%) of the younger patients were currently enrolled in school or were receiving job skills training in medically supervised professional institution. Many have less than high school education. Although four of the older patients have a high school vocational diploma (current age range 18–34 years), only one patient is physically and cognitively able to maintain part-time protected employment.

**Table 4:** Psychosocial functioning and adaptation: Scholastic level, Mental age, work activity, Psychiatric manifestations, and living arrangements

Case ID	Genotype	YOB (year)	YoHSCT (year)	Current age (years)	Best attained Scolastic Level	Mental age at last evaluation	Current activity/work status	Psychiatric manifestations		Mobility	Guardian	Living arrangement
								Status	Age at onset			
1	W402X/Q70X	1984	1986	34	^B^High school vocational degree	16	Unable to work since 2016	Depression, multiple APE	29^APE^, 30^APE^, 31^APE^ 31^D^	Able to walk	No	Home
2	Q70X/c.1190-10C>A	1982	1987	37	^CM^Primary	9–10	No activity	none		Able to walk^Dr^	Curatorship	Home
3	W402X/134del12	1987	1990	32	^B^High school vocational degree	16	Partial protected work	Depression	22^D^	Wheelchair, crutch ^Dr^	No	Home
4	W402X/W402X	1991	1993	27	^CM^Primary	9–10	Adult day care facility (unable to work)	Depression	19^D^	Wheelchair, cane, walker	Curatorship	Nursing home
5	1041_1042 del CA/1041_1042 del CA	1994	1998	24	^M^Pre-school	4	Adult day care facility (unable to work)	Depression	16^D^	Wheelchair, can walk short distance	Tutorship	Home
6	1124 del C /W402X	1994	1996	Died	^CE^Primary	7–8	Died	None		Wheelchair-bound**	Died	Died
7	W402X/Q70X	1995	1996	24	^CM^Primary	9–10	Occupational activity	Depression	20^D^	Able to walk	Curatorship	Between Home and Nursing Home
8	c.50_61del12/W402X	1995	1997	24	^CM^Primary	10–11	Unable to work	None		Wheelchair, can walk short distance	Tutorship	Home
9	W402X/W402X	1995	1997	24	^M^Pre-school	2	Unable to work	Dementia	19^DM^	Wheelchair-bound	Tutorship	Home
10	W402X/W402X	1995	1998	23	^CM^Primary	11–12	Adult day care facility (unable to work)	Depression	13^D^	Wheelchair-bound	tutorship	Nursing home
11	Q70X/W402X	1997	1998	22	^B^High school vocational degree	16	Unable to work	None		Able to walk ^Dr^	No	Lives with boyfriend
12	W402X/Q70X	1997	1998	22	^CM^Primary	10	Unable to work	Depression, hyperactivity	5-16^H^, 15^D^	Wheelchair, walker	Tutorship	Home
13	Q70X/A327P	1996	1999	22	^M^Pre-school	6	Adult day care facility (unable to work)	None		Wheelchair-bound	Tutorship	Nursing home
14	W402X/W402X	1997	1999	21	^C^Middle school	13–14	No activity	Depression	20^D^	Wheelchair, walker	Tutorship	Home
15	P533R/c.1273_1274insC	1998	2000	20	^C^Middle school	15–16	Unable to work	Depression, multiple APE	16^D^, 18^APE^,19^APE^	Able to walk	No	Home
16	W402X/W402X	2000	2001	19	^B^High school vocational degree	16	Adult day care facility	Depression	17^D^	Wheelchair, can walk short distance	No	Between home and nursing home
17	W402X/Y581X	1998	2001	20	^C^Middle school	13–14	Professionnal school	Depression, Hyperactivity, multiple APE	13^D^, 5-10^H^, 17^APE^, 19^APE^	Able to walk	Tutorship	Home
18	W402X/W402X	1999	2001	19	^CE^Primary	7	Adult day care facility (unable to work)	Hyperactivity		Wheelchair, walker	Tutorship	Home
19	Q70X/W402X	1999	2001	20	^CM^primary	14–15	Job training	None		Able to walk	Tutorship	Nursing home
20	W402X/W402X	2000	2003	19	^CM^primary	14–15	Job training	Depression	18^D^	Wheelchair, cane	Tutorship	Home
21	Q70X/W402X	2001	2002	17	^CM^primary	14–15	Adult day care facility (unable to work)	Depression	17^D^	Wheelchair, 2 canes	Curatorship	Between home and nursing home
22	W402X/W402X	2001	2004	17	^M^pre-school	5–6	Adult day care facility (unable to work)	Hyperactivity		Wheelchair, can walk short distance	Curatorship	Home
23	Q70X/c.792 + 1G > A in intron 5	2008	2009	10	^CM^primary	9	In school	None		Wheelchair, can walk short distance	NA	Home
24	ND in USA	2010	2011	9	^CE^primary	7	In school	None		able to walk,	NA	Home
25	W402X/W402X	2010	2011	8	None	2	IME*	Autism	2^A^	Able to walk	NA	Between home and nursing home

*YOB* year of birth, *YoHSCT* year of transplant; [Scholastic level completed: *M* maternelle, *CE* cours élémentaire, *CM* cours moyen, *C* collège, *B* Bac Pro]; [Mental Age as determined by speech therapist based on the best scolastic level achieved]; [current activity/work status: *IME: medico-educational institution]; [Psychiatric Manifestations: APE: acute psychotic episode; A: autism, D: depression, DM: dementia, H: hyperactivity/attention deficit disorder, none: absence of psychiatric manifestation]; [Mobility: Dr: drives a car; **prior to death]

### Psychosocial function and adaptation

Late-onset psychiatric manifestations were significant in this cohort of patients (n = 17; 68%) (Table 4). Depression was diagnosed in thirteen patients (nine females), at a median onset age of 18 years (range 13–31 years). Hyperactivity and attention deficit disorder (four males) were diagnosed at median onset age of 5 years (range 4–7 years) and treated with long-term methylphenidate or risperidone (duration of treatment 5 to 11 years). Multiple acute psychotic episodes (APE), (independent of depression) occurred in three patients at median onset age of 18 years (range 17–31 years).

On the last evaluation, none of the adult patients (n = 20) was able to live independently; a majority (75%) were living at home with parents (eight of whom had no external activities, four had occupational activities in adult daycare settings and two had a partial protected work). Seven adults were residents of nursing homes (four with occupational activities). Most, 16 (80%) adult patients required curatorship or tutorship and were incapable of legal or medical decision making.

Mobility was limited for all patients. Yet, the majority of patients (80%) could complete a 6 min Walking Test (6MWT) (albeit five of them with walking aids) with a median of 455 m (range 240–573 months) (Table 5).

**Table 5:** Onset of cardiovascular manifestations with respect to HSCT

	Before HCST				After HCST				Total
	n	(%)	Age (years)		n	(%)	Age (years)		
			Median	Range			Median	Range	N
M- dysplasia	18	72	1 year 4 months	10 months–3 year 5 months	7	28	8 years 8 months	3 years 6 months–19 years	25
A- dysplasia	10	40	1 year 4 months	11 months–2 years 10 months	11	44	7 years 10 months	2–29 years	21
M- regurgitation	14	56	1 year 4 months	10 months–3 years 5 months	11	44	4 years 5 months	2–19 years	25
A- regurgitation	2	8	1 year 6 months	1 year 4 months –1 year 8 months	14	56	11 years 7 months	3 years 6 months–21 years 11 months	16
Stenosis	0	–	–	–	6	24	15 years 6 months	7 years 5 months–21 years 11 months	6
Surgery	0	–	–	–	1	4	34 years 11 months		1

## Discussion

This cohort study confirms that despite successful HSCT engraftment, the long-term burden of disease in MPSI-H remains enormous. Benefits of successful engraftment and increased survival into the third decade of life are evident [33]. However, also observed by others, long term outcomes are poor and disease progression is evident in all [6, 28, 34–41]. We confirm that the most common clinical manifestations pre-HSCT continue to worsen included hip dysplasia, kyphosis, valvular heart disease, hearing impairment and corneal clouding [42]. This long-term follow-up study, 32 years for the oldest patient, has allowed us to observe and report not only progression of disease but also the acquisition of other undocumented disease manifestations.

The consequential poor gross and fine motor skills, compromised mobility and need for walking aids or wheelchairs, limited endurance, suboptimal breathing and persistent airway obstruction, delayed language skills, poor vision, and hearing impairment together with multiple and frequent surgical interventions are common in MPSI-H patients, all of which have negative effects on day to day functioning and the quality of life of patients (and their caregivers) [35–41].

Previously undocumented, here we report a high proportion of late-onset psychiatric manifestations (depression, psychotic episodes, hyperactivity, inattention, dementia) appearing approximately 15 years post-HSCT. During adolescence, MPS I patients often show low self-esteem, depression, and social withdrawal [10, 43]. Depression for most is still ongoing and debilitating, interfering with the patient’s psychosocial functioning and adaptation.

In France, various welfare assistance programs are available to both children and adults with disabilities (mental and physical) giving them the opportunity to live as normal a life as possible including special educational classes in the mainstream education system, as well as job training and placement services. All MPSI-H patients in this study were receiving assistance from various programs. However, despite these assistance programs, on the last evaluation, most patients could not hold down a job or function independently, and many could not make informed or independent decisions and were court-assigned curatorships and/or tutorships.

There is longstanding evidence that younger age at HSCT, full donor chimerism, and normal IDUA enzyme activity post-HSCT predict better outcomes with respect to engraftment, survival, and residual disease burden (improved somatic manifestations and cognitive function) [6, 7, 12, 16–18, 21, 25, 33, 35, 44–48]. In this study most of the transplantations occurred before the age of 30 months, but in several patients, transplantation occurred at an older age, which may have limited beneficial outcomes. Boelens et al. [11] argue that diagnosis and treatment as young as 12–24 months may already be too late as significant damage to the brain, bones and tissues has already occurred. Arguably the most effective strategy to early diagnosis and treatment is through neonatal screening and pre-symptoms treatment [21, 48].

It is commonly accepted that neurodevelopmental outcomes vary widely among transplanted MPSI-H patients [16–18, 21, 28, 38, 45, 47, 49]. Even though deterioration appeared to stabilize for most post-HSCT, and new skills were gained and learning was observed (three obtained high school vocational diplomas), most of the patients demonstrated long-term mild to moderate cognitive impairment in all domains of assessment, similar to what has been reported previously [6, 7, 12, 17, 33, 34, 36, 50, 51]. Decreased attention span and information processing in patients with MPS IH after successful HSCT may be due to decreased white matter integrity and reduction of corpus callosum volume [51]. The sample size in this study was inadequate to accurately assess correlations. Non-the-less, except for one patient whose regression was due to autism, the faster rate of regression appeared to be multifactorial and associated with two factors in one patient and three factors in three patients. The factors of interest included an age at transplant greater than 30 months, an IQ of 70 before the transplant, a heterozygous donor and a low chimerism.

In the present study, full and durable chimerism was achieved in most (76% patients), and leukocyte IDUA levels were within normal range in all patients knowing that in heterozygous donor enzyme activity was in the lower range of normal. Total urinary GAG levels were within normal ranges in all patients (except in one). However, there were abnormal fractions of heparan and dermatan sulfate present as reported by others [52], and serum IDUA levels were below normal ranges in the majority of patients despite total chimerism. It should be noted that serum IDUA activity takes years to normalize.

There are ongoing discussions at to the added benefit of supplementing HSCT with ERT, in fact a recent European consensus procedure (2011) advocated initiating ERT as early as MPS diagnosis and including those patients awaiting HSCT [14]. It has been hypothesized that ERT post-HSCT may benefit patients, and especially those in poor clinical condition prior to transplantation, or those with weak chimerism (< 50% in our opinion) by preventing complications, decreasing the risk of medical and surgical interventions required to manage disease related complications [29, 53]. ERT post-HSCT in patients with healthy homozygote donors who demonstrate full chimerism (100%) may also provide additional benefit in endurance and urinary GAG levels as suggested in a recent pilot study by Polgreen et al. [29, 53]. These results however need to be confirmed.

Some have argued that ERT supplementation should be considered on a case-by case basis, for example, to address progressive cardiopulmonary failure post-HSCT. Valayannopoulos et al. describe a case study where despite full chimerism, late infusion with ERT (12 years post-HSCT) greatly improved respiratory function which had begun to deteriorate approximately 10 years post transplantation [54].

In our study, only the few patients with low or variable chimerism post-HSCT were administered long term ERT (as it became available on the market), which normalized enzyme and urinary GAG levels. Addition of ERT post-HSCT may have benefited these patients by slowing the progression of disease in visceral organs, however, we did not observe a difference in %FVC as compared to the rest of the cohort.

Our observations indicate a largely stable pulmonary disease post-HSCT and similar to what others have reported [28, 42]. Approximately one third of patients with pre-HSCT valve involvement developed late onset stenosis and one patient developed valvuloplasty, independently of age at HSCT or IDUA enzyme activity post-HSCT as reported by others [46]. The progression of pre-existing cardiac disease may be difficult to prevent due to GAG buildup and irreversible changes in heart valve tissues [55], unless perhaps, if treated with ERT at first diagnosis [14].

Pre-HSCT ophthalmic conditions progressively continued resulting in poor visual acuity in long-term survivors despite HSCT [28, 31, 56]. As corneal injury and corneal haze are associated with myofibroblast transformations, it may be possible to prevent or slow corneal damage through complementary drugs known to prevent myofibroblast conversions [31].

Post-HSCT, hearing loss was either stabilized or normalized [6, 34], management of which still involved fitting hearing aids and repeated grommet insertions even at older ages. Most of the same patients also suffered from neurosensory hearing loss and were fitted with hearing aids that were often not well-tolerated.

Therapeutic effect of HSCT in bone pathology is not very well understood. Ossification failure and abnormal bone modeling continue despite successful transplantation, full chimerism and normal enzyme levels post transplantation, albeit at a slower rate as compared to untreated patients. We observed progressive bone manifestations (including cervical cord compressions, scoliosis, CTS, genu valgum and hip luxations) post-HSCT limiting gross and fine motor activity, and necessitating corrective orthopedic and neurosurgical interventions, also widely reported by others [23, 30, 48, 55, 57]

Benefits of concomitant, early, and aggressive surgical interventions (such as osteotomy and hip abutment for hip dysplasia, physeal stapling of genu valgum, spine fusion for thoracolumbar kyphosis, and surgical release of nerve entrapment in CTS and tendon release in trigger finger syndrome) are warranted and need to be established, especially in light of improved anesthetic techniques and knowledge of disease [26, 28, 30, 58]. In CTS, early surgical interventions can prevent muscle wasting, digital flexion contractures and subsequent reduction in hand dexterity [6, 7]. In this study, bracing therapy was attempted in all patients with thoracolumbar kyphosis for a mean duration of 8.5 years, yet in most patients posterior followed by anterior spine segment fusion was still necessary. At present there is no international consensus on the optimal surgical management of multiplex dysostosis (especially kyphosis) or whether or not all surgical interventions should be offered to all patients [59].

Compromised growth was observed in all patients having achieved adult height on the last evaluation. Linear growth is thought to be associated with age at HSCT [26]. However, we were not able to observe any difference in final height; the median adult height of the cohort presented was 142.5 cm and similar for both males and females, irrespective of age at HSCT.

### Limitations and strengths

What is unique about our study is the long and comprehensive follow-up, at the same hospital reference center and with the same multidisciplinary team of caregivers. Although we report on retrospective cohort study (observational) of successfully transplanted MPS IH patients, all along we have engaged all caregivers (physicians, therapists, and parents) in the assessment and treatment management of the multiple sequalae to improve the QOL of these patients.

Common limitations of observational cohort studies of rare diseases include the inherent small sample size, the heterogeneity of disease manifestations, and the potential for missing data. Missing or incomplete observations due to patient incapacities (cognitive impairment, behavioral attributes and poor cooperation, poor vision, impaired hearing, poor endurance and limited mobility), lack of control group, or lost archived medical charts, especially in older patients, limits robust comparison across patients. However, we present very long-term follow-up data and over 30 years of observations for some, providing rich insight into disease progression, disease management pre- and post-HSCT, and residual disease burden.

Psychosocial function was assessed using a protocol developed at the RCIMD and therefore has limited applicability. There is a need for standardized MPSI-H disease-specific tools to assess the impact of disease on quality of life and social adaptation at every stage of life, as there remain many unmet needs.

Finally, longitudinal testing and long-term follow-up of neurocognitive development has inherent limitations. As there is no one test that provides measurement of all neuro developmental domains and across all ages (from early infancy to adolescence and adulthood), it was necessary to use different tests with limited comparability and interpretability across different age groups. Furthermore, newer version of the tests may not be comparable to older versions as they may not comprise of the same domains. In this study, all adult patients were evaluated with the WAIS IV, however during the course of follow-up, several versions of the WISC were used, and domains tested, although simplified, may not have corresponded to older versions of the test.

Long-term observations documented here present a dismal and depressing progression of a fatal disease for which there is no cure, underscoring the need for development of superior therapies. The currently available treatment options have extended life expectancies and stabilized at best, but not reversed or halted progressive degenerative changes and manifestations of new late-onset symptoms.

It is important to appreciate the burden of residual disease (loss of mobility, gross and fine motor skills; below normal cognitive ability; suboptimal cardio and pulmonary function; poor vision and hearing, etc.) and the impact it has on the quality of life (patients and caregivers) and psychosocial functioning of affected individuals. Furthermore, we have observed a high prevalence of late onset psychiatric manifestations which have not been well-documented in the literature. Depression and psychotic episodes are examples of additional complications that not only have a negative effect on patient and caregiver quality of life, but also potentially interfere or impede treatment and care management of patients and must be acknowledged and dealt with.

## Conclusion

Early diagnosis and current treatment (transplantation and ERT) are warranted. Better outcomes are anticipated with homozygous donors, full chimerism and high levels of enzyme activity. Continued close and frequent follow-up of patients by a multidisciplinary team of physicians and therapists using a standardized protocol is crucial to ensure delivery of optimal and personalized care to each patient to maintain a better QOL. Standardized assessment tools are essential to accurately assess psychosocial unmet needs and to help select appropriate interventions. MPSI-H patients should be evaluated frequently as their behaviors, adaptation skills and social needs change over time, necessitating treatment adjustments accordingly. Management of orthopedic, cardiopulmonary, auditive and vision functions through timely surgical interventions can preserve mobility and endurance, and educative support and job training and integration can render some level of independence and a better QOL.

Finally, it is crucial that parents are involved in all the discussions and decision-making related to the management of MPS-IH patients. Parents need to be informed of the increasing burden of disease with age of patient.

## Methods

### Patients

Patients with MPSI-H who were successfully grafted with allogeneic HSCT between January 1986 and septembre 2011 and were alive 1-year post-transplantation were included in this study. Successfully transplanted patients underwent systematic standardized multidisciplinary follow-up evaluations on an annual basis at the RCIMD. The functionality of the graft was assessed by measuring IDUA activity in leukocyte and serum, quantitative and qualitative urinary GAG excretion and donor chimerism. Medical records of patients (before transplantation) as well as follow-up evaluations were retrospectively evaluated. Written informed consent was obtained from patients or from the parents or legal guardians of patients unable to give consent for being included in this retrospective observational study.

In all patients, diagnosis was confirmed by increased urinary excretion of dermatan and heparan sulfates as well as deficiency of IDUA activity in peripheral blood leukocytes [6]. Complete IDUA gene sequencing was performed for all patients. Transplantation was performed according to institutional protocol recommendations as previously published [7]. Transplantation outcome data for some of the patients has been reported previously [7].

### Study design

This observational study reflects retrospective analysis of Hurler patient medical records and follow-up evaluations. Patient demographic information, age at symptoms onset, date of confirmed diagnosis, date of treatment initiation, biomedical parameters related to transplantation process (donor characteristics, HLA data, conditioning regimen, GVHD), as well as date and cause of death (if patient died) were obtained from medical charts. Laboratory and imagery evaluation results, height and weight, physical examinations, date and nature of surgical interventions (if any) and other information relevant to the course of disease were record at each follow-up at the RCIMD. All patients were followed-up at the RCIMD, and the same evaluation tools and methodology was used throughout the duration of observation.

### Peri- and post-transplantation evaluation

All pre- and post-HSCT patient evaluations (neurodevelopmental, somatic and psychosocial assessments) were performed at the RCIMD. Pre-HSCT (baseline) and annual evaluations post-HSCT were conducted using a standardized protocol by a multidisciplinary team of physicians and therapists including metabolic physician, ENT specialist; ophthalmologist, audiologist, cardiologist, pneumonologist, orthopedic physician, physiotherapist, speech therapist, and psychiatrist. Chimerism monitoring was performed by a hematologist and a metabolic physician at 1 month, 3 months, 6 months, and annually thereafter post HSCT.

### Neurodevelopmental evaluation and outcome

Neurodevelopmental outcome was based on standardized and validated neurobehavioral tests at pre- and post HSCT including the Brunet-Lezine Locomotor and performance scale for patients under the age of 3 years, the Terman-Merill test for patients between ages of 3 to 6 years, and/or the nonverbal Borel Maisonny Test for those between 18 and 60 months followed by the Wechsler Intelligence Scale (WISC for children 6–16 years) and Wechsler Adult Intelligence Scale (WAIS IV for adults 16–90 years) [50]. A battery of age-specific tests for each patient aged 5 to 18 years were conducted by a speech therapist and used to evaluate scholastic level specifically comprehension, oral and written expression, reading, writing, and arithmetic. After completing this battery of tests, the speech therapist was then able to determine the functioning scholastic age of the patient, and to identify newly acquired skills. The results were compared with norms for typically developing children.

### Somatic assessments

Cardiopulmonary function tests (spirometry [FVC%], electrocardiography, echocardiography) were performed to evaluate mitral and aortic valve insufficiency, cardiomyopathy and upper respiratory obstruction. Audiologic examination (otitis, behavioral audiometry and brain stem auditory evoked responses) was performed to detect infection or neurosensory hearing loss and the need for hearing aid. Ophthalmic examination was performed to assess corneal clouding and need for corneal graft, retinopathy, glaucoma, cataracts and their interventions. Brain and spine MRI images were analyzed for abnormalities including hydrocephalus and cerebral atrophy as well as medullar compression. Orthopedic assessments (radiologic imaging and electrophysiological tests) carried out to determine need for surgical or supportive intervention including those related to kyphoscoliosis, atlantoaxial instability, genu valgum, cord compression, hip dysplasia with (sub)luxation, and carpal tunnel syndrome (CTS) and trigger finger. Growth monitoring (weight, longitudinal height, and head circumference) was done at every evaluation and results were compared to sex-specific French growth reference charts [60]. Endurance was measured when possible, using the standard 6mwt.

### Psychosocial assessment

Authors collected data on psychiatric evaluations (depression, hyperactivity, attention deficit, psychotic episodes, seizures as well as psychosocial assessment (schooling and educational level and rehabilitation interventions, as well as living situation).

### Statistics

Descriptive statistics were used to present symptoms and age at onset; age at Hurler diagnosis; age at HSCT initiation, type of transplantation, donor characteristics, chimerism and frequency of acute and chronic graft-versus-host disease (GVHD); development of somatic disease and manifestations, age at diagnosis and disease progression; developmental quotient (DQ) and age at various psychometric evaluations; and age at supportive or surgical interventions. Continuous variables are summarized as means (± std), medians and ranges. Categorical variables are summarized as frequency counts and percentages.

## Data Availability

The data generated during this study are contained in this published article.

## Abbreviations

6MWT: 6 Minutes walk test
APE: Acute psychotic episode
BMT: Bone marrow transplant
CBT: Cord blood transplant
CNS: Central nervous system
CTS: Carpal tunnel syndrome
DQ: Developmental quotient
ENT: Ear, nose, and throat
ERT: Enzyme replacement therapy
FVC (%): Forced volume vital capacity
GAG: Glycosaminoglycans
GVHD: Graft versus host disease
HLA: Human leukocyte antigen
HCT: Hematopoietic cell transplantation
HSCT: Haematopoietic stem cell transplantation
ICHT: Intracranial hypertension
IDUA: α-l-iduronidase
IQ: Intelligence quotient
MPSI-H: Mucopolysaccharidosis I severe phenotype: Hurler
MRI: Magnetic resonance imaging
PRI: Perceptual reasoning index
PSI: Processing speed index
QOL: Quality of life
RCIMD: Reference Center for Inherited Metabolic Disorders
SD: Standard deviation
VCI: Verbal comprehension index
WAIS IV: Wechsler adult intelligence scale IV
WISC: Wechsler intelligence scale for children
WMI: Working memory index
